# Hypersensitivity to Biological Treatments in Juvenile Idiopathic Arthritis: How Should It Be Managed?

**DOI:** 10.3390/jcm11247291

**Published:** 2022-12-08

**Authors:** Muserref Kasap Cuceoglu, Ozge Basaran, Ozge Soyer, Seza Ozen

**Affiliations:** 1 Division of Pediatric Rheumatology, Department of Pediatrics, Hacettepe University, Ankara 06100, Turkey; 2 Division of Allergy, Department of Pediatrics, Hacettepe University, Ankara 06100, Turkey

**Keywords:** juvenile idiopathic arthritis, biological treatments, hypersensitivity reactions, anaphylaxis, desensitization

## Abstract

Juvenile idiopathic arthritis (JIA) is one of the most frequent diseases in the practice of pediatric rheumatology. JIA treatments have been modified and improved with the use of biological drugs along with technological innovations. However, different types of hypersensitivity reactions to biological drugs have also been reported. Anaphylaxis and infusion reactions occurring during the intravenous infusion require a critical approach in the acute period. On the other hand, the detection of drug-related late-type reactions and the development of antibodies to the agent highlight the need for an understanding of the drug-induced etiology to prevent the patient from continuing the treatment with the culprit drug. The chronic disease process, concomitant immune dysregulation, and multiple drug use may result in these hypersensitivity reactions. In this review, the hypersensitivity reactions to the biological treatments used in patients with juvenile idiopathic arthritis and the management of these conditions are discussed.

## 1. Introduction

Juvenile idiopathic arthritis (JIA) is the most common childhood rheumatic disease around the world. The treatment aims to cure the disease, ensure the quality of life of the patients and prevent comorbidities. The use of biological therapies has increased in recent years [1]. Biologics are targeted against cytokines (e.g., IL-1, TNF), receptors (e.g., IL-6R, CD28), signaling molecules (e.g., JAKs) and cells (e.g., B cells). Their structures may be in the form of monoclonal antibodies, fusion proteins, naturally occurring molecules or small molecule inhibitors. There are four types of biologics that are commonly used in JIA, namely tumor necrosis factor-α (TNF) inhibitors (infliximab, adalimumab, etanercept), B-cell inhibitors (rituximab (anti-CD-20)), interleukin inhibitors (anakinra, canakinumab, secukinumab, tocilizumab) and selective co-stimulation modulators (abatacept) [2,3]. 

Biological drugs are targeted therapies, making them superior to other drugs. Although highly efficient, biological drugs can cause various hypersensitivity reactions, including infusion-related reactions (IRRs), cytokine release reactions (CRRs), type I reactions (IgE/non-IgE) and mixed reactions [4]. Most drugs are small compounds with molecular weights of less than 1 kDa. On the other hand, biological drugs are larger-sized proteins designed to be structurally similar to structural proteins with molecular weights much greater than 1 kDa [5]. Thus, a great range of hypersensitivity reactions are to be expected with the use of these drugs. In this review, we aimed to evaluate the hypersensitivity reactions to biological drugs used in JIA and to summarize the recommendations for management. We must underline that we often do not know the exact mechanism of hypersensitivity with these drugs. Furthermore, one may suggest that any drug may cause any type of reaction. However, we have reviewed the relevant publications and attempted to categorize the hypersensitivity reactions for simplification. For categorization, we relied on the report of the European Academy of Allergy and Clinical Immunology (EAACI) in 2021 (Table 1) [4].

## 2. Hypersensitivity Reactions 

**Figure 1:**
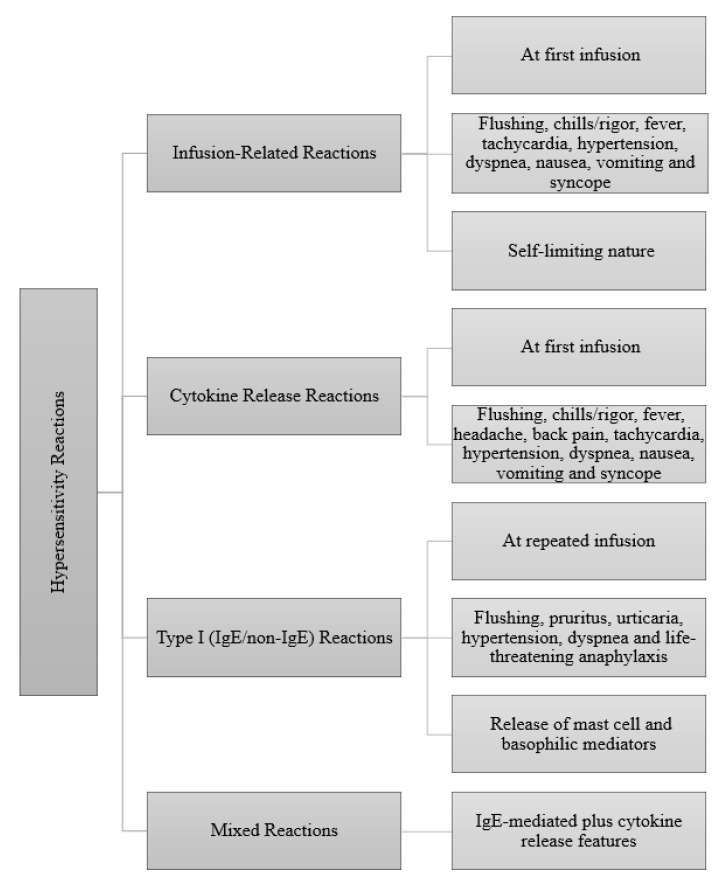
Clinical presentations of immediate reactions to biologics [4].

The hypersensitivity reactions (HSRs) are characterized as either immediate (<1h–6 h) or non-immediate or delayed (>6 h)) reactions [6]. The factors affecting these reactions include the type of immunoglobulin (IgE, Ig G, Ig M), complement activation, degree of humanization of the monoclonal antibody and adjuvants or excipients. The hypersensitivity reactions associated with biologics relate to reports of delayed-type HSRs, including serum-sickness-like symptoms, rash, vasculitis, toxic epidermal necrolysis, Stevens–Johnson syndrome (SJS) and drug reaction with eosinophilia and systemic symptoms (DRESS) [7]. Immediate-type HSRs include cytokine release syndrome (CRS), acute infusion-related reactions and Ig E-mediated hypersensitivity reactions (anaphylaxis) (Figure 1). The most common two immediate reactions are IgE/non-IgE-mediated reactions (63%) and infusion-related reactions (IRRs) (50–20%). Another type of immediate reaction is CRS (13%). The most important feature distinguishing acute infusion reactions from anaphylactic-type reactions is that they can occur by mechanisms other than Ig E-mediated reactions and are predictable and preventable. Therefore, acute infusion reactions in most patients can be prevented by infusing the same agent at a slower rate and administering it with premedication [8,9]. 

### 2.1. Acute Infusion-Related Reactions 

Common acute infusion reactions constitute a significant number of reactions to monoclonal antibodies. Usually, these reactions can occur with the first dose. The related clinical findings include fever, flushing, rash, tachycardia, hypertension, dyspnea, back pain, vomiting and syncope. Currently, the pathogenesis of these reactions is unknown [10]. The release of proinflammatory cytokines may play a role in some reactions, which are predictable, common and usually mild. The activation of the complement system is also thought to play a role in immediate hypersensitivity reactions, since C3a and C5a may directly stimulate mast cells. Mast cells may also play a role in the pathogenesis due to the fact that the first dose of the drug used in an acute infusion reaction distinguishes it from the Ig E-mediated reaction that occurs with re-exposure to the antigen [10].

Management: Mild-to-moderate infusion reactions can be managed by slowing the infusion rate and via premedication with corticosteroids, antihistamines and antipyretics.

Which biological drugs have been reported with this reaction? Infliximab [11], rituximab and tocilizumab [12].

### 2.2. Cytokine Release Syndrome

**Figure 2:**
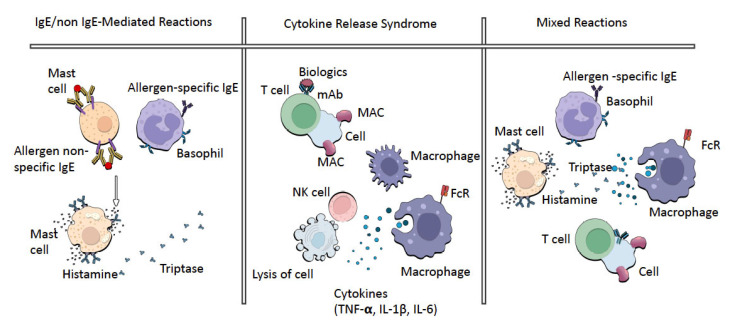
The pathogenesis of immediate HSRs.

Cytokine release syndrome (CRS) is a reaction characterized by hypotension or hypertension, rash, nausea, headache, tachycardia and hypoxia. The clinical signs and symptoms are usually due to cytokine release. CRS is associated with TNF-α, interferon-γ and interleukin-6, released by monocytes, macrophages, cytotoxic T cells and NK cells (Figure 2). Tests can show elevated serum TNF-α and IL-6 levels at the time of the reaction when compared with their normal baseline levels. In CRS, flu-like symptoms to severe conditions (cardiac dysfunction, pulmonary edema, renal failure and death) have been documented [13]. In this sense, it is a rather scary condition. The main difference between IRRs and CRRs is the self-limiting nature of the IRRs upon repeated exposure and the response to premedication [4].

Management: For mild reactions in CRS, the management approach includes premedication with corticosteroids and acetaminophen and slowing the infusion rate. Severe reactions in CRS are managed as anaphylaxis [13]. Tocilizumab is administered in moderate or severe cytokine release syndrome undergoing CAR-T cell therapy. The most important reason for this is that IL-6 plays a key role compared to the cytokines released in CRS, as mentioned above [14,15].

Which biological drugs have been reported with this reaction? Infliximab and rituximab.

### 2.3. Ig E-Mediated Hypersensitivity Reactions

IgE-related hypersensitivity reactions occur with basophil and mast cell activation. IgE is released by mediators, basophils and mast cells, which play a role in the pathogenesis of IgE-mediated HSR compounds such as histamine, platelet-activating factor, leukotrienes, tryptase and prostaglandins. The systemic clinical findings include urticaria, angiedema, flushing, cough, dyspnea, nausea, vomiting and hypotension. In addition, elevated serum levels of tryptase are helpful for the differential diagnosis of IgE-mediated reactions. Anaphylaxis (IgE-mediated hypersensitivity) is defined as a severe life-threatening systemic hypersensitivity reaction. A large number of cases with anaphylaxis induced by biological drugs (tocilizumab, etanercept, etc.) have been reported [16,17]. Soyer et al. have suggested a certain desensitization protocol against biological hypersensitivities, especially for tocilizumab and rituximab in JIA patients [17,18] (Supplementary Table S1). The first dose of infliximab can cause anaphylaxis, although this is an exception because IgE-related reactions occur after the sensitization phase with repeated exposure to drugs [19].

**Figure 3:**
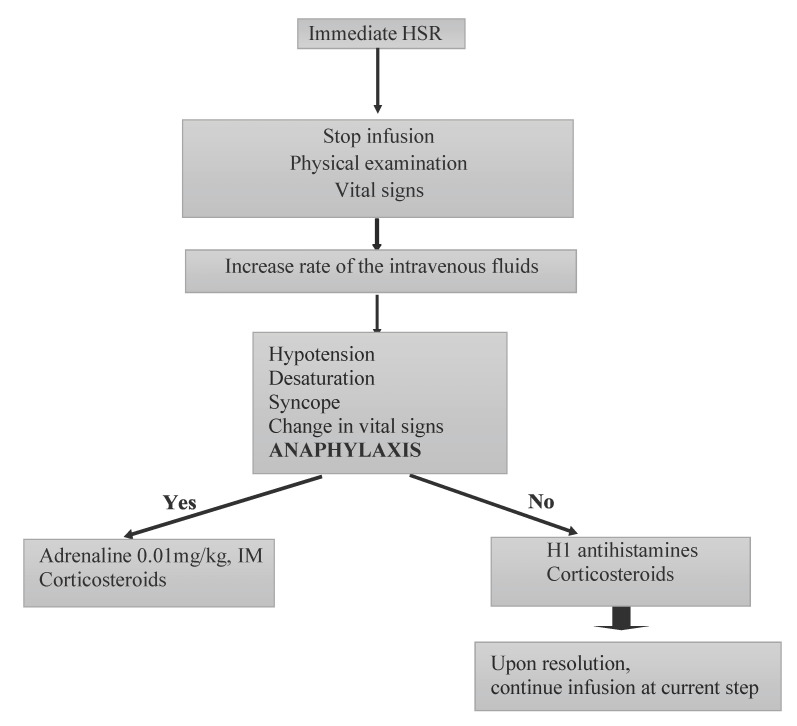
The algorithm for the management of the immediate type of hypersensitivity reactions with biologics. This figure was adapted from Ref. [22]. HSR: hypersensitivity reactions; IM: intramuscular.

Management: When anaphylaxis occurs, the first step should be the administration of adrenaline intramuscularly, after the assessment of vital signs and immediate cessation of the infusion. If the patient does not respond to adrenaline within 5–10 min, another dose of intramuscular adrenaline should be given. The second and third steps should consist of administering oxygen, nebulized adrenaline, nebulized beta-2-agonist, normal intravenous saline, corticosteroids, antihistamines and intravenous adrenaline if necessary [20] (Figure 3).

The measurement of serum tryptase levels within 30–120 min after the reaction is required to better distinguish anaphylaxis from other hypersensitivity reactions. Higher serum tryptase levels relative to baseline are associated with mast cell activation, although sometimes the serum tryptase levels may be detected into the normal range in anaphylaxis. In the anaphylaxis against monoclonal antibodies, the mast cells temporarily do not respond to the allergen during skin tests for 4 weeks. Therefore, prick skin testing with the culprit agent should be performed 4–6 weeks after the reaction. No standard protocol has been defined for biological drugs other than adalimumab, etanercept and infliximab. Recently, different skin test concentrations for abatacept, rituximab and tocilizumab have been reported in the literature in some cases [18]. Desensitization should be considered after an evaluation of the risks and benefits of the culprit biological drug.

Which biological drugs have been reported with this reaction? Adalimumab, etanercept, infliximab, abatacept, rituximab, anakinra [21] and tocilizumab [18].

### 2.4. Delayed Generalized Cutaneous Symptoms

Delayed cutaneous symptoms present with subcutaneous injections and are characterized by erythema, swelling and itching 1–2 days following the injection.

Management: These reactions are limited to symptomatic treatment with ice, oral antihistamines and topical corticosteroids [23].

Which biological drugs have been reported with this reaction? This reaction is commonly associated with TNFs (etanercept and adalimumab) and anakinra, and usually occurs within the first two months of therapy.

### 2.5. Drug Reaction with Eosinophilia and Systemic Symptoms (DRESS)

Drug reaction with eosinophilia and systemic symptoms (DRESS) is a severe late-type hypersensitivity reaction characterized by a generalized skin rash with visceral organ involvement, lymphadenopathy, eosinophilia and atypical lymphocytosis. The clinic features may vary, and the course of the disease is typically prolonged. Occasionally, disease flare ups may continue to occur despite the discontinuation of the drug causing the problem. It typically takes two to eight weeks from the beginning of the therapy to the onset of DRESS [24].

Management: In systemic JIA, Saper et al. have reported the association with the HLA DRB1*15 haplotype [25]. Delayed drug reaction with eosinophilia and systemic symptoms (DRESS) has been reported in patients with systemic JIA receiving interleukin (IL)-1 or IL-6 inhibitors (anakinra, canakinumab, rilonacept and tocilizumab) [26]; thus these researchers recommend an assessment of the presence of this haplotype for individuals before starting biological drugs. If DRESS occurs, the treatment is to discontinue the culprit drug and apply other DMARDs; case reports have suggested switching to cyclosporine, intravenous immunoglobulin (IVIG), corticosteroid and JAK inhibitors [27,28]. When the patient is in clinical remission for MAS, a change of medication may be considered. However, this is not a recommendation that has been agreed on, and many centers do continue IL1 and IL6 blockers. These biologics are life-saving medications, and each case should be considered individually [29,30,31,32,33,34].

Which biological drugs have been reported with this reaction? Anakinra, canakinumab, rilonacept and tocilizumab.

### 2.6. Acute Generalized Exanthematous Pustulosis (AGEP)

Acute generalized exanthematous pustulosis (AGEP) is a rare drug-related hypersensitivity reaction [35]. The acute eruption is characterized by the development of sterile pustules on edematous erythema. Fever and peripheral blood leukocytosis are usually present. AGEP is 90% drug-related.

Which biological drugs have been reported with this reaction? Infliximab, etanercept and adalimumab.

Although not reported in JIA patients, there are a few case reports of AGEP developed with anti-TNF-alpha agents (etanercept, adalimumab) in psoriasis [36,37,38]. On the other hand, there are also a few papers on the effective use of infliximab and secukinumab in the treatment of AGEP [39].

### 2.7. Toxic Epidermal Necrolysis (TEN)/Stevens–Johnson Syndrome (SJS)

Stevens–Johnson syndrome (SJS) (in which skin detachment is <10 percent of the body surface) and TEN (skin detachment of >30 percent of the body surface area) are severe mucocutaneous reactions, most commonly triggered by medications characterized by extensive necrosis and detachment of the epidermis [40]. The mucous membranes are affected in the patients. To date, there are no case reports of SJS/TEN triggered by biological drugs used in juvenile idiopathic arthritis.

Which biological drugs have been reported with this reaction? Rituximab.

There are no case reports of SJS/TEN triggered by biological drugs used in juvenile idiopathic arthritis, but TEN related to RTX in patients with lymphoma has been reported [41].

## 3. Desensitization

Desensitization is defined as the temporary clinical tolerance to the offending drug. Desensitization should be planned and managed by pediatric allergists. Relevant protocols have been published elsewhere [22].

However, some steps can be summarized: Desensitization is indicated in patients who have previously experienced type I or type IV HSRs (except for severe cutaneous adverse reactions) to the culprit drug. However, desensitization is contraindicated in the following conditions: type II reactions (immunocytotoxic reactions), vasculitis, type III reactions (serum-disease-like syndrome) and serious cutaneous adverse reactions (SJS/TEN, DRESS, AGEP). Antihistamines, systemic corticosteroids, montelukast and acetaminophen can be used as premedications due to the symptomology of the index reaction before desensitization. The breakthrough reactions during desensitization episodes are treated accordingly. For the subsequent desensitization episodes, the desensitization protocol is modified by adding an additional step at two steps backwards to slow the infusion rate, and additional premedications are given unless the patients do not receive them. If a breakthrough reaction still occurs during desensitization despite these procedures, a reduction in the total targeted dose or alternative medications for the primary disease may be considered. In addition, the normal prophylactic saline infusion can be used to prevent CRS during desensitization.

## 4. Conclusions

Biological drugs have the potential to stimulate the immune system due to their therapeutic properties. The underlying disease is also an important factor in the development of HSRs. Additionally, Soyer et al. reported that renal involvement, frequent hospitalizations and exposure to more than two different biological drugs are risk factors for HSRs with biologics in children with rheumatologic disease [17].

On the other hand, immediate HSRs are known to be associated with antidrug antibodies (ADAs). Essentially, it is more accurate to use human anti-chimeric antibodies (HACA) for infliximab and human antihuman antibodies (HAHA) for other agents. For patients receiving infliximab with other immunosuppressants, the incidence of HACA is lower, whereas infliximab applications at longer intervals increase the risk of developing HACA. It should be noted that cross-reactivity among TNF-α inhibitors is not common, so the patient could be switched to other TNF-α inhibitors such as adalimumab and etanercept.

In conclusion, biological therapies are highly effective treatments for the current management of JIA, as well as other inflammatory and autoimmune conditions. With their increased use, unexpected side effects are likely to occur. The recognition and treatment of immediate and delayed-type hypersensitivity reactions to biological drugs are important for the rheumatologist. In addition, it should be kept in mind that hypersensitivity reactions secondary to biological drugs should be considered by an expert pediatric allergist. A multidisciplinary approach is crucial to safely maintain appropriate treatments for JIA patients.

## Figures and Tables

**Table 1:** Hypersensitivity reactions to biological treatments according to their clinical presentations [4] and management.

	Acute Infusion-Related Reactions	Cytokine Release Syndrome	IgE-Mediated Hypersensitivity Reactions/ Anaphylaxis	Delayed Generalized Cutaneous Symptoms	Drug Reaction with Eosinophilia and Systemic Symptoms (DRESS)	Acute Generalized Exanthematous Pustulosis (AGEP)	Toxic Epidermal Necrolysis (TEN)/ Stevens–Johnson Syndrome (SJS)
Anakinra							
Canakinumab							
Anti-TNF							
Etanercept							
İnfliximab							
Adalimumab							
Tocilizumab							
Secukinumab							
Abatacept							
Rituximab							*
Management	Premedication (corticosteroids, antihistamines, antipyretics) and slow the infusion rate	Mild reactions: Slow the infusion rate (or a break) and premedication with corticosteroids and acetaminophen Severe reactions: Treat as an anaphylaxis	Discontinue the culprit drug, treat the acute reaction according to the severity and consider desensitization	Symptomatic treatment with ice, oral antihistamine and topical corticosteroids	Discontinue the culprit drug and switch to other DMARDs	Discontinue the culprit drug and switch to DMARDs	Discontinue the culprit drug and switch to DMARDs

* TEN related to rituximab in patients with lymphoma has been reported. Note: Grey areas indicate the specified type of reactions occur with these drugs. The HSRs mentioned in the table only include previously reported articles.

## Supplementary Materials

The following supporting information can be downloaded at: https://www.mdpi.com/article/10.3390/jcm11247291/s1, Table S1: An example protocol for tocilizumab desensitization.
