# Supplementary material for: Hypersensitivity to Biological Treatments in Juvenile Idiopathic Arthritis: How Should It Be Managed?
Source: J Clin Med. 2022 Dec 8;11(24):7291. doi: 10.3390/jcm11247291 (PMC9785630; doi:10.3390/jcm11247291)
Supplement: Supplementary file 1 [file jcm-11-07291-s001.zip › Supplementary,Table S1.pdf]

Table S1. An example protocol for tocilizumab desensitization.

Step	Solution	Time (min)	Total infusion time	Volume per infused step (mL)	Dose administered/min(mg/min)	Dose administered / step (mg)	Cumulative dose (mg)
1	1	15	15	0.6	0.0004	0.006	0.006
2	1	15	30	1.2	0.0008	0.012	0.018
3	1	15	45	2.4	0.0016	0.024	0.042
4	1	15	60	4.8	0.0032	0.048	0.09
5	2	15	75	1	0.0064	0.096	0.186
6	2	15	90	2	0.0128	0.192	0.378
7	2	15	105	4	0.0256	0.384	0.762
8	2	15	120	4.5	0.03	0.45	1.212
9	2	15	135	6	0.04	0.6	1.812
10	2	15	150	9	0.06	0.9	2.712
11	2	15	165	12	0.08	1.2	3.912
12	2	15	180	15	0.1	1.5	5.412
13	2	15	195	30	0.2	3	8.412
14	2	15	210	45	0.3	4.5	12.9
15	3	475	685	200	0.4	190	200

Solution 1 = 1 mg tocilizumab + 100 mL serum physiologic (0.01 mg/mL). Solution 2 = 10 mg tocilizumab + 100 mL serum physiologic (0.1 mg/mL). Solution 3 = 190 mgtocilizumab + 200 mL serum physiologic (0.95 mg/mL)
